# Study design with responsible return of results for a fully remote genome sequencing study in individuals with Prader-Willi syndrome

**DOI:** 10.1016/j.gimo.2025.103448

**Published:** 2025-08-11

**Authors:** Caroline J. Vrana-Diaz, Jessica Bohonowych, Jaimie L. Richards, Brandon M. Wilk, Manavalan Gajapathy, Yael Bar-Peled, Anna C. Harris, Jessica J. Denton, Donna Brown, Elizabeth A. Worthey, Theresa V. Strong

**Affiliations:** 1 Foundation for Prader-Willi Research, Covina, CA; 2 Division of General Internal Medicine and Population Science, University of Alabama at Birmingham Heersink School of Medicine, Birmingham, AL; 3 Center for Computational Genomics and Data Science, Department of Genetics, University of Alabama at Birmingham Heersink School of Medicine, Birmingham, AL; 4 Department of Clinical and Diagnostic Sciences, University of Alabama at Birmingham, Birmingham, AL; 5 Department of Genetics, University of Alabama at Birmingham Heersink School of Medicine, Birmingham, AL

**Keywords:** Genome sequencing, Patient advocacy group, Pharmacogenomics, Prader-Willi syndrome, Secondary findings

## Abstract

**Purpose:**

Prader-Willi syndrome (PWS) is a complex neurodevelopmental genetic disorder affecting multiple systems. We describe the design and feasibility of a fully remote, patient group-led, genome sequencing (GS) study that will evaluate the impact of genetic variants on the frequency and severity of PWS clinical symptoms, with responsible return of results.

**Methods:**

A total of 50 participants, or their legally authorized representative, provided consent via videoconference discussion and selected which genetic results would be returned. Participants were sent dried blood spot cards for GS and a buccal swab kit for orthogonal pharmacogenomics analysis. A subset of legally authorized representative LARs participated in semistructured interviews about their GS experience.

**Results:**

All 50 participants completed the study and elected to receive their primary findings, and 48 of 49 participants who consented to receive pharmacogenomic information returned the buccal swab kit. Forty-seven participants consented to receive secondary findings per current American College of Medical Genetics guidelines; 2 had actionable results, with online genetic counseling support provided. Three families received medically significant findings related to variants associated with blood clot formation, which is important because individuals with PWS are at an increased risk for thrombotic events. Interview participants expressed a high degree of interest in the findings and emphasized the ease of participating in the study but felt the process was lengthy.

**Conclusion:**

A fully remote GS study is feasible to perform within a rare disease population, and responsibly returning genetic findings that are important to families is achievable.

## Introduction

Prader-Willi syndrome (PWS) is a rare genetic neurodevelopmental disorder affecting multiple organ systems, with an estimated prevalence between 1 in 10,000 to 1 in 30,000 individuals (OMIM 176270).^1^, ^2^, ^3^, ^4^ PWS results from a lack of expression of the paternally expressed, imprinted genes within the PWS critical region on chromosome 15q11.2-q13.^2^ Between 60% and 70% of PWS cases are due to a deletion within the PWS critical region on the paternally inherited chromosome 15. These are further classified as type 1 deletions (involving breakpoints BP1 and BP3) or the smaller type 2 deletions (involving breakpoints BP2 and BP3).^2^^,^^5^ The next most common genetic subtype (35%-40%) is uniparental disomy of the maternal chromosome 15, which can be further classified into complete isodisomy, segmental isodisomy, or heterodisomy. Another 3% to 5% of PWS cases are due to an imprinting center defect on the paternal PWS critical region by either pathogenic DNA variant, deletion, or epimutation, and chromosomal translocations can cause PWS in rare cases.^2^^,^^6^

PWS is characterized by a complex phenotype that changes over the lifespan. In early infancy, PWS is associated with poor appetite, feeding difficulties, failure to thrive, and extreme hypotonia.^1^, ^2^, ^3^, ^4^ However, in early childhood, the poor appetite diminishes and hyperphagia (an unrelenting appetite and lack of satiety) emerges, leading to morbid obesity if the food environment is not strictly controlled.^7^ Additional manifestations of PWS include developmental delays and cognitive challenges, hypogonadism, growth hormone deficiency, decreased pain sensitivity, reduced gastrointestinal motility, strabismus, scoliosis, sleep abnormalities, as well as an increased risk of mental illness, seizures, and thrombotic events.^1^^,^^2^^,^^4^^,^^8^^,^^9^ Individuals with PWS typically present with some degree of intellectual disability and exhibit a characteristic behavioral profile that includes temper outbursts, heightened anxiousness, repetitive behaviors and questioning, cognitive rigidity, oppositional behavior, and social cognition deficits.^10^

Both the clinical symptoms and the behavioral phenotype in PWS occur with varying degrees of frequency and severity among individuals. Certain features are common, such as hyperphagia and growth hormone deficiency (90%-100% of individuals), whereas other features are less common (maladaptive behaviors: 70%-90%, skin picking: 50%-60%, scoliosis: 40%-80%, strabismus: 40%-60%, and seizures: 10%-20%).^2^ The Global PWS Registry has been instrumental in better characterizing the clinical and behavioral features of PWS,^7^, ^8^, ^9^^,^^11^, ^12^, ^13^, ^14^, ^15^, ^16^, ^17^ but the individual risk factors for these symptoms remain unclear. One potential explanation for symptom variability could be the presence of variants throughout the genome that influence the expression of certain PWS characteristics. Because genome sequencing (GS) is not necessary to diagnose PWS given the accuracy of existing, less resource-intensive methods, such as DNA methylation analysis,^2^ clinical GS is typically not offered to individuals with PWS. Thus, the impact of DNA variants outside the PWS region on the clinical and behavioral characteristics of the disorder has not been explored to date.

Because PWS is a rare disorder with a challenging behavioral profile, participation in clinical research can place a significant strain on families already burdened by the disorder. Challenges for clinical research participation include travel to a study site for clinic visits, time away from other family members/responsibilities, and financial burden associated with participating.^18^ To address the gaps in knowledge with respect to how genetic variants across the genome may affect the phenotypic variability seen in PWS, a fully remote GS study of individuals with PWS was designed. This pilot study, led by a patient advocacy group, prioritized reducing the burden on participation, as well as the responsible return of actionable genetic information of interest to families. A description of this study is presented, including the design and implementation of the fully remote study, and return of pharmacogenomics (PGx) results and secondary findings important to participants, with resources to support their interpretation. Qualitative interviews with the legally authorized representative (LAR) (typically, a parent) assessed the process and utility of the study. The main genetic findings from this remote GS study will be reported separately.

## Materials and Methods

Participants were recruited via the Global PWS Registry,^11^ focusing on those interested in being contacted for additional research opportunities and residing in the United States. The Global PWS Registry was launched in 2015 under the National Organization of Rare Disorders IAMRARE Registry Platform^11^ and is managed by the Foundation for Prader-Willi Research. Participants were included if they were between the ages of 10 and 65, had genetically confirmed PWS, had a Global PWS Registry record that was complete or updated within 6 months of the study start date, and were able to give blood via a dried blood spot (DBS) card. The North Star independent nonprofit research ethics review board reviewed and approved the protocol, consents, and participant-facing materials (NB100013).

The entire study was performed remotely to reduce burden, including remote consenting. The consenting process was performed over videoconference, led by the study Principal Investigator and the study coordinator, with the participant and/or the LAR given ample time to ensure all questions were answered before signing the informed consent and assent documents. In some cases, assent for the participant with PWS was waived because of age or intellectual maturity, which was determined by the participant’s LAR. During remote consent, participants (or their LAR) had additional choices during the consenting process. They were able to consent to receive their pharmacogenomic information, their actionable secondary findings, and/or their unanalyzed GS data. They also selected how their data might be shared in future studies. These options included (1) no data sharing and no future contact, (2) can be recontacted for consent to individual future studies, or (3) data sharing with any or all of the following: researchers at the University of Alabama at Birmingham (UAB), the sponsoring organization (Foundation for Prader-Willi Research), other universities, the National Institutes of Health (NIH), or pharmaceutical companies.

**Figure 1:**
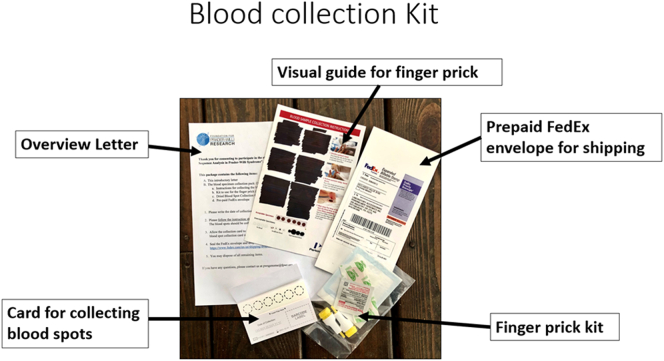
**Materials included in the dried bloodspot collection kit sent to participants and caregivers for genome sequencing.**

After consenting to participate in the study and selecting the type of results they would like to receive and with whom these results could be shared, participants were mailed a blood collection kit from PerkinElmer Genomics (now Revvity Omics). For the GS analysis, the blood sample was collected at home by the caregiver of the participant with PWS using a finger prick and spotting blood on a DBS card. Participants were provided with all necessary materials, including an informational letter with step-by-step instructions, a finger prick kit (including two lancets, an antiseptic wipe, and Band-Aid), a DBS collection card, a tip sheet for a successful finger prick, a video and picture instructions on how to open and use the finger prick lancet, and a pre-paid FedEx envelope to send the DBS card back to PerkinElmer Genomics for sequencing (Figure 1).

Participants opting to receive pharmacogenomic information required orthogonal DNA analysis before results were returned. They were mailed a buccal swab kit from Kailos Genetics, and the sample was collected at home by the caregiver. All materials needed for buccal cell collection were sent to participants, including a welcome letter, step-by-step instructions, 2 cheek swabs, clear envelope to place the completed swabs with a silica gel packet, and a prepaid USPS return envelope to send the swabs back to Kailos Genetics for analysis. The PGx testing panel also included high-risk variants in the prothrombin (*F2;* HGNC:3535), and Factor V (*F5*, Leiden variant; HGNC:3542) genes, which predispose individuals to blood clots.

Phenotypic information was collected through caregiver-reported surveys housed within the Global PWS Registry. This information included details on demographics (age, sex, height, weight, and genetic subtype), congenital defects, sleep, seizures, endocrine history, orthopedics, vision, thrombosis history, behavior, mental health, psychiatric medications, and hyperphagia and food behaviors. Genetic diagnosis of PWS was documented for all participants.

### Analytic approach, data management, and data protection

GS was performed at PerkinElmer Genomics (now Revvity). Sequence reads were returned to the Computational Genomics and Data Science Center at UAB where they were analyzed by the Genomic Analysis Team according to current best practices. In brief, sequence reads were aligned to the GRCh38 reference genome with BWA-MEM and small variants (single nucleotide, insertions, deletions, and insertion-deletions) were called using Haplotypecaller following GATK best practices.^19^^,^^20^ Quality control for reads, alignments and variant calls was conducted using QuaC. Structural variants (SV), regions of homozygosity, and mosaicism were identified using Manta,^21^ AutoMap,^22^ and B-allele frequency calculations (Holt J, Birch C, Brown D, et al. Programmatic detection of diploid-triploid mixoploidy via whole genome sequencing*. bioRxiv.* 2018.), respectively. Variants from each individual were analyzed using the clinically validated software application Codicem.^23^ Analysts employed multiple filtering strategies as previously described to identify rare and more common variants. SV analysis was performed as previously described (Holt JM, Birch CL, Brown DM, et al. Identification of pathogenic structural variants in rare disease patients through genome sequencing. *bioRxiv.* 2019:627661).^24^ Variants were classified into pathogenicity categories, according to the American College of Medical Genetics and Genomics (ACMG) guidelines.^25^ The DNA sequence of each potentially returnable variant was validated by clinical-grade Sanger Sequencing performed at Revvity Genomics. A Variant Review Committee, which consisted of members of the Genomic Analysis Team and additional scientists with relevant expertise, reviewed and acted on all potentially returnable variants. Potential returnable variants included any variants identified as potentially disease-causing, additional variants thought to contribute to the participant’s disease phenotype, and any secondary findings, including ACMG-recommended actionable secondary variants, as well as variants that may be medically significant for the participant.

### Return of results

Participants received a report of their primary results, which included the GS analysis outcome focused on the PWS region of chromosome 15, via secured email.

Additionally, participants could consent to receive a PGx report via secure email, detailing DNA variants that may influence medication response as determined by orthogonal DNA analysis by Kailos Genetics. Finally, participants could consent to the return of secondary findings. These secondary findings were reported following the guidelines set by the ACMG, which suggest reporting on variants considered to be actionable (ie, a medical result in which actions can be taken to prevent, screen, reduce, or treat symptoms of the health condition). At the time of reporting, that list included 73 actionable genes (ACMG SF v3.0) related to cancer, cardiovascular, inborn errors of metabolism, and miscellaneous phenotypes.^26^ The authors collaborated with My Gene Counsel, a digital health company that provides personalized, easy-to understand, digital genetic counseling reports via their Living Lab Reports solution^27^ to generate a report specific for this study. As per the consent discussion, the participant and LAR identified a physician familiar with the care of the individual with PWS to participate in the return of results discussion regarding actionable secondary findings.

### Qualitative interviews

LARs were invited by email to participate in a qualitative interview to share their overall experience with the study. Interview questions were collaboratively developed by members of the research team. Interviews were conducted by a genetic counseling student through a Health Insurance Portability and Accountability Act secure Zoom meeting and were recorded and transcribed. Transcripts were reviewed for accuracy before coding and analysis using NVivo (version 15.0.1). Two members of the research team coded the transcripts independently utilizing an inductive approach. After reaching a consensus on codes most relevant to the research, themes and subthemes were developed by reflexive thematic analysis.

## Results

**Figure 2:**
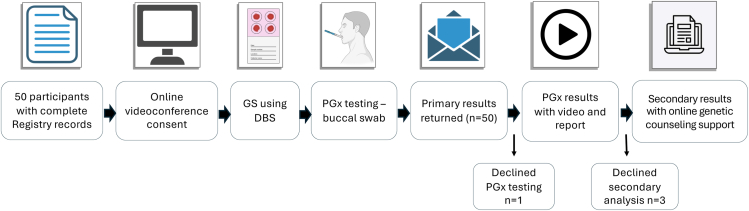
**Project overview**. Individuals with complete records in the Global PWS Registry consented with their LAR via teleconference. Dried blood spots were used for GS, with buccal swabs for orthogonal PGx testing. Return of results included video and online support to assist with interpretation of the reporting. DBS, dried blood spot; GS, genome sequencing; LAR, legally authorized representative; PGx, pharmacogenomics; PWS, Prader-Willi syndrome.

An overview of the project flow is depicted in Figure 2. Fifty participants were enrolled in this study. The demographic information of the participants is detailed in Table 1. Participants ranged in age from 10 to 47 with a mean age of 19.9 years (SD of 9.7) and came from 27 different states throughout the United States. The cohort was approximately equal with respect to sex, and the genetic subtype distribution was consistent with what has been reported in the literature.

**Table 1:** Demographics of the participants with PWS in this genome sequence analysis study (*N* = 50)

Demographics	*N* (%)
Sex	
Female	28 (56%)
Male	22 (44%)
Mean age ± SD	19.99 ± 9.7
Age range	10-47
Race	
White	46 (92%)
Non-White	4 (8%)
Global PWS Registry-reported PWS genetic subtype	
Deletion	28 (56%)
Uniparental disomy (UPD)	18 (36%)
Imprinting defect (ID)/don’t know/other	4 (8%)
Mean BMI ± SD	25.3 ± 7.5
BMI range	15.8-46.1

The informed consent documented the participant’s preferences on receiving pharmacogenomic information, actionable secondary findings per the current recommendations of the ACMG,^26^ and/or their entire unannotated/interpreted GS data (Table 2). The consent also provided the option to consent to use their genetic information in additional PWS-related studies, either through blanket approval or via reconsent for future studies. Most participants consented to receive pharmacogenomic information and actionable secondary findings, whereas 18% of participants chose to receive their entire GS data (Table 2). In terms of future data sharing, the majority of participants consented to some level of sharing (with the most common data sharing exception being that of industry partners); 18% of participants chose to be recontacted to consent to individual opportunities, and none opted for no data sharing or recontact (Table 2).

**Table 2:** Consenting and data sharing preferences of participants (*N* = 50)

Preferences	*N* (%)
Consenting preferences	
Pharmacogenomic information	49 (98%)
Actionable secondary findings	47 (94%)
Raw genome sequencing data	9 (18%)
Future data sharing preferences	
No data sharing and no recontact	0 (0%)
Recontact for individual research opportunities	9 (18%)
Consented to share with researchers at FPWR/UAB	41 (82%)
Consented to share with researchers at other universities	34 (68%)
Consented to share with the NIH	34 (68%)
Consented to share with industry partners	29 (58%)

*FPWR*, Foundation for Prader-Willi Research; *NIH*, National Institutes of Health; *UAB*, University of Alabama at Birmingham.

All blood spot cards (*N* = 50) were received by PerkinElmer Genomics for GS. Two blood spot cards were unable to be sequenced by PerkinElmer because of insufficient blood spotted on the cards; therefore, replacement blood spot cards were sent to those participants, both were returned, and the GS was successfully completed. The primary reports detailing the GS results of the participant’s PWS region of chromosome 15 were sent to all participants (*N* = 50). These results included the location of any SV. These variants include deletions, runs of homozygosity, which are indicative of uniparental disomy or an absence of these SV (deletions/runs of homozygosity), that could indicate uniparental disomy with heterodisomy or an epimutation within the PWS imprinting center.^5^ In-depth research findings from the DNA results will be reported in a follow-up article.

Because the use of psychiatric medication is common in individuals with PWS and pharmacogenomic information is of interest to the PWS community,^28^ participants were given the option of receiving a PGx report detailing how their DNA variants affected drug metabolism. One participant declined PGx results. For the remaining 49 participants, buccal swab kits were sent to Kailos Genetics for orthogonal analysis of pharmacogenomic findings from GS. All Kailos PGx results were congruent with the participant’s respective GS. Two of the original 49 kits could not be analyzed because of sample quality issues; therefore, replacement buccal swab kits were sent, and both were successfully completed. In total, 48 of the 49 kits were returned, resulting in 48 PGx reports being provided to participants.

**Figure 3:**
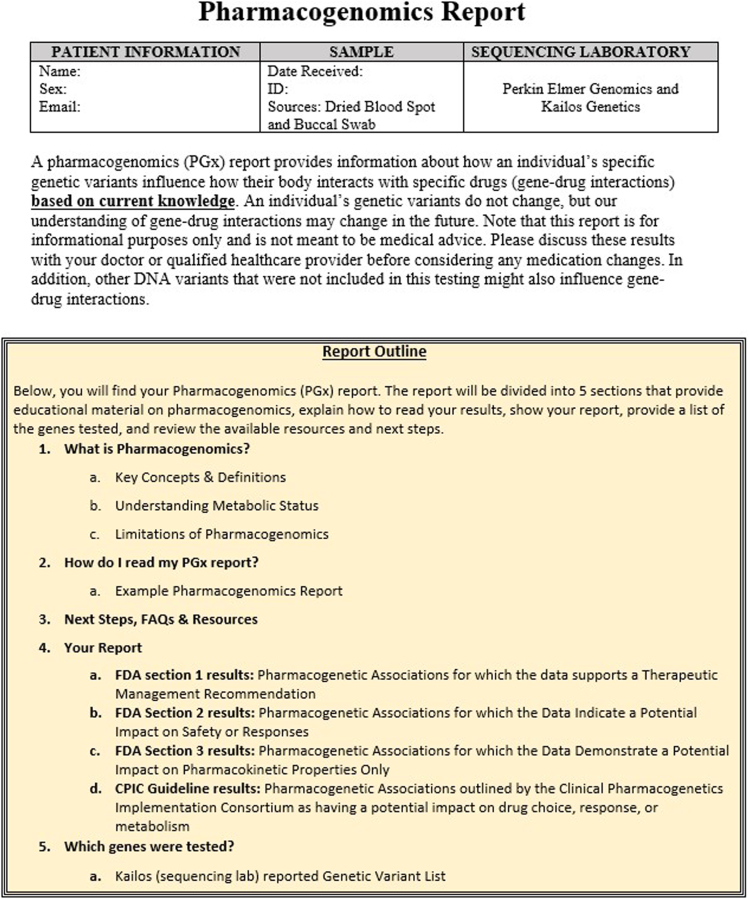
**Table of contents of the pharmacogenomics report returned to participants, detailing its main sections**.

The PGx report was developed with the input of a PWS Genomics Community Board, a consultant trained in the responsible return of genomic data, and a genetic counseling student along with their advisor at UAB. The PGx report contained the guidelines of both the US Food and Drug Administration (FDA),^29^ as well as the Clinical Pharmacogenomics Implementation Consortium (CPIC).^30^ This report synthesized the information returned from the orthogonal PGx report from Kailos Genetics into a report that could be readily comprehended by participants and their families. The PWS Genomics Community Board provided input into the layout of the sample report, provided feedback on what types of resources they would need to understand the results, and approved the final PGx report template. Figure 3 details the table of contents of the PGx report returned to participants who consented to receive this information, and a full sample report can be found in the Supplemental Report 1. Given the complexity of the PGx results, a brief (∼15-minute) video “walk through” of the PGx report, discussing how the results were presented and where to find additional information, was developed and shared with families before their receipt of the PGx report. Additional surveys assessing the utility of the PGx information were sent to participants before receiving their PGx report, 1-month after return of results, and 6-months after return of results. Forty of the 48 participants (83%) completed the 1-month after return of results survey, and 39 of the 48 participants (81%) completed the 6-month survey. The manuscript detailing the results of the preresults survey has been published elsewhere,^28^ and the postreturn survey findings will be reported separately.

The PGx panel included detection of DNA variants associated with increased risk of thrombosis. Two participants were found to carry high-risk variants in the *F2* gene (HGNC:3535), and 1 participant had a high-risk Leiden variant in the *F5* gene (HGNC:3542). Given that these variants increase risk for blood clots, and individuals with PWS are at higher risk for thrombosis than the general population,^9^^,^^31^^,^^32^ additional information from the National Library of Medicine regarding the risk associated with the variants was included in the PGx report for these individuals.^33^^,^^34^

The secondary findings analysis was only performed in participants who consented to receive these results (*N* = 47). Of these 47, 2 participants (4.3%) were found to have secondary findings deemed as actionable by the ACMG-73 list,^26^ consistent with the 2% to 3% that has been previously reported.^35^ A pathogenic variant in the *BRCA2* gene (HGNC:1101) was detected in one participant, and a likely pathogenic variant in the *CACNA1S* gene (HGNC:1397) was detected in another participant. These findings were reported to the participant and/or the LAR, in consultation with a physician identified by the participant and/or LAR to be involved in the secondary findings discussion, and with additional online support information through My Gene Counsel. Individuals who had no reportable variants on the ACMG list received a report documenting negative findings.

LARs were invited to participate in a semistructured interview to understand their perception of the GS process and return of results. Nine LARs participated in the interviews, which were recorded and used to generate themes and subthemes. Table 3 shows the themes and representative quotes from the LARs who participated in qualitative interviews. Major themes that emerged regarding motivations to participate included a desire for more information and an interest in research. With respect to secondary findings, LARs expressed how this information helped them feel better prepared for their loved one’s future. Participants also found the genetic reports informative and, in many cases, reassuring. There were a small number of participants whose GS results differed from the parent-reported original diagnosis results. One participant reported a type 1 deletion, but GS showed a type 2 deletion, and 2 reported type 2 deletions, but GS showed type 1 deletions (one was a self-report, and the other was a genetic report based on early chromosomal array analysis). The overall perception of the GS experience was positive, with participants commenting on the ease of participating; however, participants noted the extended time it took to receive all the genetic results.

**Table 3:** Themes, subthemes, and representative quotes from qualitative interviews of LARs

Theme	Subthemes	Representative Quote
Motivations to participate	Interest in research Desire for more information	“I will say it was helpful because it got me more aware of deeper studies and where things are going with Prader-Willi testing.” – LAR 1 “And I'm also excited that it is a living data bank and as new things are associated that the genome can be revisited in order to see if there have been new things uncovered that may make a difference in her life.” – LAR 4 “Just more info, the better educated we’ll be.” – LAR 9
Secondary findings	No secondary findings found Secondary findings found Preparing for the future	“Well, I was happy to find out that she didn't have any of these markers of the 73 genes that are recognized as actionable, so I was thrilled about that.” – LAR 3 “There was one that there was some information that she may be susceptible to more blood clotting… Yeah, in fact my son then got tested for that same [condition].” – LAR 6 “Because you're not just what your 15th chromosome say[s] you are. There are a lot of other things out there that modern medicine is developing that could be good to know when it comes time to evaluate health problems.” – LAR 4 “You know, sometimes with illnesses, the signs and symptoms are more subtle or could be overlooked, and I think we deal with a lot of things as a parent to somebody with Prader Willi, a lot of complex medical issues, and it would be very easy, in my opinion, to miss something that could be happening because you're concentrating or focused on maybe another medical need at the moment. So yeah, for me, it was just about having that knowledge, so I could be prepared.” – LAR 5
Report outcomes	Understanding the report Verification/reassurance Surprising results	“I overall found it [the report] very helpful.” – LAR 8 “Well, I mean it, just it confirmed that she was a UPD PWS person, which we kind of what we already knew that… So it was basically just a confirmation of what I already knew.” – LAR 3 “I was surprised to find out that the description of her deletion is different than the description that we have been given in the past by other places who run DNA testing, but not genomic testing.” – LAR 4
Overall process with GS	Sample collection Process length Ease of process	“The first blood sampling was a bit tough with a Prader-Willi kid.” – LAR 7 “I would say, if there was an answer that I wanted quickly this would not be the test to do it on because it felt like it took a really long time.” – LAR 2 “A bit slow in getting reports out, but I understand that that took some time, because we had to, you know, have them reviewed, and also, you know, make sure that the counseling and the support was there.” – LAR 7 “Participation was straightforward. The information on how to complete the process was clear. The instructions were clear.” – LAR 5 “It's super non-invasive, right? I mean, here's some blood which our kids have to give all the time, not always the easiest thing, but it's super easy and just to have all of that information is priceless.” – LAR 8

*GS*, genome sequencing; *LAR*, legally authorized representative.

## Discussion

The overall goal of this GS study is to identify molecular variants affecting the phenotype and/or treatment response in individuals with PWS. Given the rarity of PWS and the burden of traveling to study sites, we sought to evaluate the feasibility of conducting a fully remote genomics-based study that prioritized the responsible return of genetic information to families who might be unduly burdened by the traditional in-clinic GS model.

This study relied on a collaborative process with participants to reduce burden and increase the likelihood of successful acquisition of biological samples in the home. Based on caregiver feedback, we created additional participant-facing materials. For blood spot collection, these included a video detailing how to open and use the finger prick lancet, and additional PWS-specific blood collection tips because people with PWS often have poor circulation in their fingers. Given the unique challenges in sample collection for individuals with PWS (eg, poor peripheral circulation and thick, viscous saliva^2^), the kit included additional instructions to rinse the mouth and increase the wait time before buccal cell collection. With this additional instruction, the process of obtaining adequate biological samples for GS and orthogonal PGx testing was feasible. The caregivers who participated in the qualitative interviews appreciated the clear instructions and the noninvasive nature of sample collection (albeit with challenges related to PWS) and found the supporting materials helpful (Table 3).

Special considerations were needed to responsibly return results (both pharmacogenomic results and secondary findings) to participants without a genetic counselor present. These considerations included a PGx report, developed with the input of a community advisory panel, genetic counselor, and genetic counseling student, which contained definitions of potentially confusing terms, the limitations of PGx testing, and links to resources for participants for any needed follow-ups. Furthermore, the participants and LARs were given the option during the consenting process to decline their pharmacogenomic report or their actionable secondary findings report. Notably, there was a strong desire in study participants to receive secondary genetic information. Only 1 of 50 (2%) individuals declined the return of the PGx report, and 3 of 50 (6%) individuals declined the return of actionable secondary findings. For those participants who declined the return of secondary findings, the Genomic Analysis Team at UAB did not search for any potential actionable secondary findings for those specific participants to prevent ethical dilemmas if any variants were found. The high level of interest in the return of genetic results in this study corroborates a systematic review of 44 studies of stakeholder views on secondary findings (SF) that found “an overwhelming majority of stakeholders believe that some form of SF should be returned if identified.”^36^ The LARs who participated in the qualitative interviews echoed this sentiment and were excited about the knowledge gained from these reports (Table 3). A Living Lab Report, accessible via the internet, was returned to all participants who consented to receive secondary findings, either as a research-grade negative secondary findings report, or a medical-grade positive secondary findings report.

An additional consideration was made for this population in the return of special variants with the PGx results. Individuals with PWS have an elevated risk of blood clots compared with the general population,^9^^,^^35^ and the Kailos PGx panel included 2 high-risk variants (in Prothrombin (F2) and Factor V (F5) genes) that predispose individuals to blood clots. This information was deemed important to return to participants, and additional information on the interpretation of these variants was included within the collaborative PGx report for those participants who were positive for high-risk variants.

Overall, patient advocacy groups (PAGs) are becoming more proactive in directing research, rather than being simply passive participants. Genome sequencing studies have been of interest to the PWS community for some time; however, the rarity of the condition and the challenges of travel to large academic centers have limited opportunities to conduct GS analysis. As a patient community with a robust patient Registry, a PAG-led remote GS offers a unique opportunity to address research questions about phenotypic variability of PWS, while providing participating families with genetic information that might be directly applicable to their clinical care. Therefore, we gathered the appropriate collaborators to successfully implement this GS study. The involvement of a PAG facilitated several aspects of the study, including recruitment to the study, providing a platform for up-to-date phenotypic data collection, making changes to the sample collection protocol in response to participant feedback, and incorporating the patient perspective into the reporting of genetic findings through a community advisory board.

For this GS study, there were many successes and challenges. GS is no longer cost prohibitive, but some aspects of this study were quite labor and time intensive, including the consenting process and report creation. We created a slide deck to explain all aspects of the study to go with the consent discussion, including the sample collection process, receiving secondary findings, receiving PGx results, and future data sharing, all considering differing levels of education and knowledge regarding GS among the caregivers and participants. Similarly, primary and secondary genetic reports, as well as a PGx report template and informational video were developed by the team with input from the patient community. Feedback from the community was essential to this process; the PGx report “walk through” video was developed when it became clear that the complexity of the PGx findings did not lend itself to comprehension based only on a written report.

Additional time intensive processes were the data analysis, gathering the Genomic Analysis Team to discuss and decide what results were reported back to families, and returning the secondary results to the caregiver and participant, with the support of their physician. The caregivers who participated in the qualitative interview portion of this study commented on the length of this process but understood the many steps needed for report return (Table 3). Including a physician familiar with the PWS participant in the discussion of secondary findings also proved challenging in some cases because the physicians did not necessarily have prior knowledge of the study. Additional resources to support these physicians might facilitate these interactions. In addition, given the expanding use of GS in the clinical setting, resources from other initiatives (eg, the All of Us program, (https://allofus.nih.gov/) may facilitate the development of accessible information adapted to specific rare disease communities.

There were several limitations of this study. The participant population was restricted to those who lived in the United States, and the study included only English-speaking participants, due to the limited nature of the resources for this study. Any planned future studies will work to expand the catchment area to additional countries and include translations of study materials to additional languages. This also may increase minority and underrepresented group participation, which could address the lack of representation of minority participants seen in our study. Additionally, participants were drawn from the Global PWS Registry, which has a relative overrepresentation of college-educated individuals. An expanded study would need to ensure that accompanying resources are appropriate for a broad range of educational levels. This recruitment approach also resulted in clinical phenotypes that were precollected and caregiver reported. Although there may be concerns that caregiver-reported clinical information is not as accurate as clinician-reported information, to date, data from the Global PWS Registry have aligned with clinical reporting.^8^^,^^15^ Finally, this study used a separate specimen collection and testing pipeline to independently confirm PGx results. Other studies have used more efficient methods by returning PGx information as part of the GS results, but challenges in calling some PGx variants led us to favor orthogonal confirmation.

### Conclusion

Overall, our study finds that a fully remote blood spot-based GS study is feasible in a rare disease population. This approach can enhance accessibility and convenience for patients with rare diseases by allowing them to participate from home, regardless of location or transportation challenges. A community-based, collaborative process can help to broaden the participant group, increase engagement, and foster a more patient-centered research process. Close collaboration with the patient community is imperative to reduce burden, increase the chances of success, and ensure that genetic information important to families is responsibly returned.

## Data Availability

Data from this genome sequencing study will be made available upon request to those trained in human subjects research. If you would like to make a request for data access, please email theresa.strong@fpwr.org.

## Conflict of Interest

The authors declare no conflicts of interest.
