# Supplementary material for: Study design with responsible return of results for a fully remote genome sequencing study in individuals with Prader-Willi syndrome
Source: Genet Med Open. 2025 Aug 11;4:103448. doi: 10.1016/j.gimo.2025.103448 (PMC13156615; doi:10.1016/j.gimo.2025.103448)
Supplement: Supplemental Material [file mmc1.pdf]

Pharmacogenomics Report

PATIENT INFORMATION	SAMPLE	SEQUENCING LABORATORY
Name:<br>Sex:<br>Email:	Date Received:<br>ID:<br>Sources: Dried Blood Spot and Buccal Swab	Perkin Elmer Genomics and Kailos Genetics

A pharmacogenomics (PGx) report provides information about how an individual's specific genetic variants influence how their body interacts with specific drugs (gene-drug interactions) **based on current knowledge**. An individual's genetic variants do not change, but our understanding of gene-drug interactions may change in the future. Note that this report is for informational purposes only and is not meant to be medical advice. Please discuss these results with your doctor or qualified healthcare provider before considering any medication changes. In addition, other DNA variants that were not included in this testing might also influence gene-drug interactions.

Report Outline

Below, you will find your Pharmacogenomics (PGx) report. The report will be divided into 5 sections that provide educational material on pharmacogenomics, explain how to read your results, show your report, provide a list of the genes tested, and review the available resources and next steps.

1. **What is Pharmacogenomics?**
   a. Key Concepts & Definitions
   b. Understanding Metabolic Status
   c. Limitations of Pharmacogenomics
2. **How do I read my PGx report?**
   a. Example Pharmacogenomics Report
3. **Next Steps, FAQs & Resources**
4. **Your Report**
   a. FDA section 1 results: Pharmacogenetic Associations for which the data supports a Therapeutic Management Recommendation
   b. FDA Section 2 results: Pharmacogenetic Associations for which the Data Indicate a Potential Impact on Safety or Responses
   c. FDA Section 3 results: Pharmacogenetic Associations for which the Data Demonstrate a Potential Impact on Pharmacokinetic Properties Only
   d. CPIC Guideline results: Pharmacogenetic Associations outlined by the Clinical Pharmacogenetics Implementation Consortium as having a potential impact on drug choice, response, or metabolism
5. **Which genes were tested?**
   a. Kailos (sequencing lab) reported Genetic Variant List

A. Key Concepts & Definitions

Clinical Pharmacogenetics Implementation Consortium (CPIC): CPIC is an international consortium of individual volunteers and a small, dedicated staff who are interested in facilitating use of pharmacogenetic tests for patient care.

Food and Drug Administration (FDA): The FDA is responsible for protecting the public health by ensuring the safety, efficacy, and security of human and veterinary drugs, biological products, and medical devices; and by ensuring the safety of our nation's food supply, cosmetics, and products that emit radiation.

Gene: refers to a short section of genetic information, or DNA. A gene has a specific job to perform in the body. There are two copies of every gene, one from the biological mother and one from the biological father. Most of the genes described in a PGx report are important for processing drugs in the body.

Genetic Variant: refers to a difference in the DNA sequence that makes up a gene in one person compared to another. Genetic variants may impact metabolism.

Genotype: refers to the two versions of a specific gene inherited from an individual's biological parents.

Metabolism: refers to the body's ability to process drugs.

Pharmacogenomics (PGx): refers to the study of how differences in our DNA can impact how we respond to certain drugs (gene-drug interaction). Everyone has differences in their DNA called "genetic variants" and some of these variants can help doctors and scientists predict how we may respond to certain medications. These variants can tell doctors how fast or slow our bodies may process a drug or how our immune systems may react to a drug.

Pharmacokinetics: refers to the study of how drugs move within the body, including the time course of the absorption, availability, distribution, metabolism, and excretion of the drug.

Phenotype: refers to the expression of the genotype which, along with the environment, contributes to an individual's observable traits or functions, such as how quickly an individual can metabolize a specific drug.

B. Understanding Metabolizer Type

The ***table below*** can be used as a reference to better understand how the drug-gene interactions in your report impact your ability to metabolize certain drugs.

Metabolizer Type	Activity	Drug Impact
**Poor Metabolizer (PM), Intermediate Metabolizer (IM)**<br>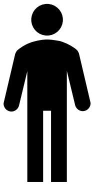	Decreased	Depending on how the drug works in the body,<br>▪ *The drug is less effective at the standard dose*<br>***OR***<br>▪ *Has a higher chance of side effects*<br>Drug dose change or changing drug may be recommended.
**Normal Metabolizer (NM)**<br>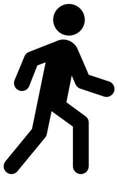	Normal	Normal, drug likely works as it should.
**Rapid Metabolizer (RM), Ultra-Rapid Metabolizer (UM)**<br>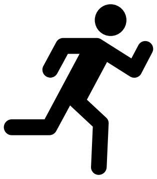	Increased	Depending on how the drug works in the body,<br>▪ *The drug is less effective at the standard dose*<br>***OR***<br>▪ *Has a higher chance of side effects*<br>Drug dose change or changing drug may be recommended.

C. Limitations of Pharmacogenomics

1. PGx information should be reviewed by healthcare providers

PGx information is one of many factors considered when prescribing drugs. A healthcare provider may decide not to change the drugs a person is currently taking based on PGx results because:

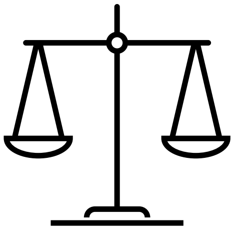

- The benefits of a current drug are greater than the potential risks of taking a new drug.
- The benefits of a current drug are greater than not taking any drug at all.
- Based on current health status, a particular drug is not required.
- However, this PGx information may be helpful to know in the future if specific health concerns arise and new drugs are suggested.

2. PGx information is limited

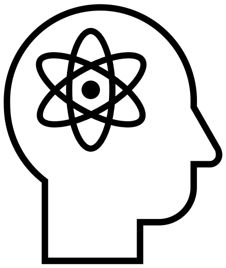

- The list of known drug-gene interactions is continually growing.
- A PGx report captures these known interactions at the current time.
- New drug-gene interactions may be discovered in the future and guidelines will be updated periodically as new genetic variants are studied.

How do I Read My PGx Report?

A. Example Report: **for demonstration purposes only*

Drug (Brand Name(s))	Drug Class(es)	Description of Gene-Drug Interaction	Phenotype	Genotype
Drug X (Brand Name X)	Immuno-suppressant	Results in lower systemic concentrations, lower probability of achieving target concentrations and may result in higher rejection risk.	Intermediate Metabolizer	Variant*1/ Variant*2

Drug (Brand Name(s)): Drugs with a high level of evidence of altered metabolism in people with certain genetic variants

Drug Class(es): Type of drug

Description of Gene-Drug Interaction: Description of the Gene-Drug interaction from the Food and Drug Administration (FDA) and/or the Clinical Pharmacogenomics Implementation Consortium (CPIC).

Phenotype: Your metabolizer type (does not change)

Genotype: Your genotype (does not change)

Within the description of gene-drug interaction, the Food and Drug Administration (FDA) has three categories (Sections 1-3) based on the level of evidence as of September 1st, 2022.

- **FDA Section 1** guidelines have the most evidence and provide a description of specific gene-drug interactions along with drug/dosage recommendations.
- **FDA Section 2** guidelines have less evidence than Section 1 and provide a description of specific gene-drug interaction but may or may not have drug/dosage recommendations.
- **FDA Section 3** guidelines have the least evidence and provide a description of the potential gene-drug interaction that may impact pharmacokinetic properties only, but the impact of these genetic variants on the safety or response of the corresponding drug has not been established

Clinical Pharmacogenomics Implementation Consortium (CPIC) guidelines include a standard system for grading levels of evidence, prescribing recommendations based on genotype/phenotype, and a standard system for assigning strength to each prescribing recommendation.

Next Steps, FAQs, & Resources

Next Steps

1. **Talk with your healthcare provider:**
   - Note that this report is for informational purposes only and is not meant to be medical advice. Please discuss these results with your doctor or qualified healthcare provider before considering any medication changes.
   - Review current drug(s) taken and discuss how the DNA variants described in this report may impact drug efficacy, side effects, and management.
   - Consider whether the results of this report support change(s) in drug(s) taken.
   - In some situations, this information will be helpful for future use; for example, in determining whether a new drug may be beneficial.

2. **Ask Questions:**

   Frequently Asked Questions for healthcare providers:
   - What might these PGx results mean for my child's current or future healthcare?
   - How can we add these PGx results to my child's electronic medical record?
   - Will this office update me of any new drug guidelines as they become available?
   - How can we use these PGx results when researching clinical trials my child is eligible for?

Healthcare Providers Who Can Help Interpret Results

1. **Pharmacogenomic Genetic Counselors**
   - Pharmacogenomic genetic counselors have training in both medical genetics and counseling. They can help interpret pharmacogenomic report results and explore patient options.
   - Find a Genetic Counselor (in person and telehealth options available)
     - https://findageneticcounselor.nsgc.org
   - If you choose to have counseling, *you will be financially responsible for that service.*

2. **A Trusted Medical Doctor or Pharmacist**

Patient-Friendly Resources

Pharmacogenomics basics with videos

- **Mayo Clinic**
  - https://www.mayo.edu/research/centers-programs/center-individualized-medicine/patient-care/pharmacogenomics
- **NIH**
  - https://www.genome.gov/dna-day/15-ways/pharmacogenomics
- **Medline Plus**
  - https://medlineplus.gov/genetics/understanding/genomicresearch/pharmacogenomics
- **National Institute of General Medical Sciences**
  - https://www.nigms.nih.gov/education/pages/factsheet-pharmacogenomics.aspx

Resources for Healthcare Providers

Specific variant guidelines and recommendations (updated frequently)

- **Clinical Pharmacogenetics Implementation Consortium (CPIC) Guidelines**
  - https://cpicpgx.org/guidelines/
- **Food and Drug Administration (FDA) Table of Pharmacogenetic Associations**
  - https://www.fda.gov/medical-devices/precision-medicine/table-pharmacogenetic-associations
- **Consider consultation with a CPIC Member**
  - https://cpicpgx.org/members/

PAGE INTENTIONALLY LEFT BLANK. YOUR PHARMACOGENOMICS REPORT IS BELOW

Your Report

PATIENT INFORMATION	SAMPLE	SEQUENCING LABORATORY
Name:<br>Sex:<br>Email:	Date Received<br>ID:<br>Sources: Dried Blood Spot and Buccal Swab	Perkin Elmer Genomics and Kailos Genetics

Genetic Variants with <u>Known Impacts</u> on Drug Metabolism

The table below includes a list of the genetic variants with ***known impacts*** (those with scientifically sufficient evidence) on drug metabolism identified in [FirstName LastName]. As of September 1st, 2022, these specific genetic variants have been noted by the US Food and Drug Administration (FDA) and the Clinical Pharmacogenetics Implementation Consortium (CPIC) Guidelines as affecting drug metabolism with a high level of evidence. There are 3 sections within the FDA reported associations: **Section 1)** Pharmacogenetic Associations for which the Data Support Therapeutic Management Recommendations; **Section 2)** Pharmacogenetic Associations for which the Data Indicate a Potential Impact on Safety or Response; and **Section 3)** Pharmacogenetic Associations for which the Data Demonstrate a Potential Impact on Pharmacokinetic Properties Only.

- This information ***does not*** replace medical advice.
- It is ***strongly recommended*** that you consult with a healthcare provider who has access to the individual's complete medical history before making any drug changes.
- Please see *Resources for Healthcare Providers* in the **Resources** page (below) for the most up-to-date information
- See the **Key Concepts & Definitions** page (above) for definitions

Results as of September 1st, 2022

FDA Section 1: Pharmacogenetic Associations for which the Data Support Therapeutic Management Recommendations

Drug (Brand Name(s))	Drug Class(es)	Description of Gene-Drug Interaction	Phenotype	Genotype
Brivaracetam (Briviact)	anticonvulsant	Results in higher systemic concentrations and higher adverse reaction risk.	Intermediate Metabolizer	CYP2C19*1/*2
Clobazam (Onfi, Sympazan)	benzodiazepine	Results in higher systemic active metabolite concentrations. Dosage adjustment is recommended. Refer to FDA labeling for specific dosing recommendations.	Intermediate Metabolizer	CYP2C19*1/*2
Clopidogrel (Plavix)	antiplatelet agent	Results in lower systemic active metabolite concentrations, lower antiplatelet response, and may result in higher cardiovascular risk. Consider use of another platelet P2Y12 inhibitor.	Intermediate Metabolizer	CYP2C19*1/*2
Azathioprine (Imuran, Azasan)	immunosuppressant	Alters systemic active metabolite concentration and dosage requirements. Results in higher adverse reaction risk (myelosuppression). Dosage reduction is recommended in intermediate metabolizers for TPMT. Intermediate metabolizers for both genes may require more substantial dosage reductions. Refer to FDA labeling for specific dosing recommendations.	Intermediate Metabolizer	TPMT *1/ *3A
Mercaptopurine (Purinethol)	purine antagonist	Alters systemic active metabolite concentration and dosage requirements. Results in higher adverse reaction risk (myelosuppression). Intermediate metabolizers may require dosage reductions based on tolerability. Intermediate metabolizers for both genes may require more substantial dosage reductions. Refer to FDA labeling for specific dosing recommendations.	Intermediate Metabolizer	TPMT *1/ *3A
Thioguanine (Tabloid)	purine analog	Alters systemic active metabolite concentration and dosage requirements. Results in higher adverse reaction risk (myelosuppression). Intermediate metabolizers may require dosage reductions based on tolerability. Intermediate metabolizers for both genes may require more substantial dosage reductions. Refer to FDA labeling for specific dosing recommendations.	Intermediate Metabolizer	TPMT *1/ TPMT *3A

FDA Section 2: Pharmacogenetic Associations for which the Data Indicate a Potential Impact on Safety or Response

Drug (Brand Name(s)	Drug Class(es)	Description of Gene-Drug Interaction	Phenotype	Genotype
Voriconazole (Vfend)	triazole	Results in higher systemic concentrations and may result in higher adverse reaction risk.	Intermediate Metabolizer	CYP2C19*1/*2

FDA Section 3: Pharmacogenetic Associations for which the Data Demonstrate a Potential Impact on Pharmacokinetic Properties Only

Drug (Brand Name(s)	Drug Class(es)	Description of Gene-Drug Interaction	Phenotype	Genotype
Dexlansoprazole (Dexilant)	proton pump inhibitor	Results in higher systemic concentrations.	Intermediate Metabolizer	CYP2C19*1/*2
Doxepin (Silenor)	tricyclic antidepressant	Results in higher systemic concentrations.	Intermediate Metabolizer	CYP2C19*1/*2
Escitalopram (Lexapro)	SSRI	May alter systemic concentrations.	Intermediate Metabolizer	CYP2C19*1/*2
Omeprazole (Prilosec)	proton pump inhibitor	Results in higher systemic concentrations.	Intermediate Metabolizer	CYP2C19*1/*2

CPIC Guidelines

Drug (Brand Name(s)	Drug Class(es)	Description of Gene-Drug Interaction	Phenotype	Genotype
peginterferon alfa-2a (Pegasys) (PegIntron), peginterferon alfa-2b, ribavirin (Rebetol)	interferons	**Implications for PEG-IFN-α and RBV therapy:** Approximately 30% chance of sustained virologic response (defined by undetectable serum viral RNA 12–24 weeks after the end of treatment) after 48 weeks of treatment for Hepatitis C infection. Consider implications before initiating PEG-IFNα- and RBV-containing regimens. **Implications for protease inhibitor combinations with PEG-IFN-α and RBV therapy**: Approximately 60% chance of sustained virologic response after 24–48 weeks of treatment for Hepatitis C infection. Approximately 50% of patients are eligible for shortened therapy regimens (24–28 weeks). Consider implications before initiating PEG-IFN-α- and RBV-containing regimens.	Unfavorable Response	IFNL3: rs12979860:C/T

Which Genes Were Tested?

A. Your Kailos-reported Genetic Variant List and Genotyping Results

All genetic variants identified from Kailos Genetics are listed in the next few pages. New information about how these specific genetic variants impact drug metabolism may be available in the future. Reviewing this list every 2-3 years may be beneficial. Participants may seek new analyses on their pharmacogenomic information at their own initiative. In addition, other DNA variants that were not included in this testing might also influence gene-drug interaction.

Please see links in the Resources for Healthcare Providers section of the **Resources** page for the most up-to-date information.

kailos

PGx genes assayed at Kailos Genetics

ABCB1	EDN1
ABCG2	F2
ADRA2A	F5
ADRB1	GNB3
AGT	GRIK4
CACNA1C	HTR1A
CES1	HTR2A
CFTR	HTR2C
COMT	IFNL3
CYP1A2	KCNIP1
CYP2C9	LDLR
CYP2C19	MTHFR
CYP2D6	NR1H3
CYP3A4	OPRM1
CYP3A5	RYR1
DPYD	SL6A2
DRD1	SLCO1B1
DRD2	TPMT
DRD3	VKORC1
